# Identification of differentially expressed non-coding RNAs in the plasma of women with preterm birth

**DOI:** 10.1080/15476286.2024.2449278

**Published:** 2025-01-13

**Authors:** Waqasuddin Khan, Samiah Kanwar, Mohammad Mohsin Mannan, Furqan Kabir, Naveed Iqbal, Mehdia Nadeem Rajab Ali, Syeda Rehana Zia, Sharmeen Mian, Fatima Aziz, Sahrish Muneer, Adil Kalam, Akram Hussain, Iqra Javed, Muhammad Farrukh Qazi, Javairia Khalid, Muhammad Imran Nisar, Fyezah Jehan

**Affiliations:** a Biorepository and Omics Research Group, Department of Pediatrics and Child Health, Faculty of Health Sciences, Medical College, The Aga Khan University, Karachi, Pakistan; b Infectious Diseases Research Lab (IDRL), Department of Pediatrics and Child Health, Faculty of Health Sciences, Medical College, The Aga Khan University, Karachi, Pakistan

**Keywords:** Preterm birth, Cf-RNA-seq, ncRNAs, TAC assay, annotations

## Abstract

This study aimed to identify differentially expressed non-coding RNAs (ncRNAs) associated with preterm birth (PTB) and determine biological pathways being influenced in the context of PTB. We processed cell-free RNA sequencing data and identified seventeen differentially expressed (DE) ncRNAs that could be involved in the onset of PTB. Per the validation via customized RT-qPCR, the recorded variations in expressions of eleven ncRNAs were concordant with the *in-silico* analyses. The results of this study provide insights into the role of DE ncRNAs and their impact on pregnancy-related biological pathways that could lead to PTB. Further studies are required to elucidate the precise mechanisms by which these DE ncRNAs contribute to adverse pregnancy outcomes (APOs) and their potential as diagnostic biomarkers.

## GRAPHICAL ABSTRACT

**Figure:**
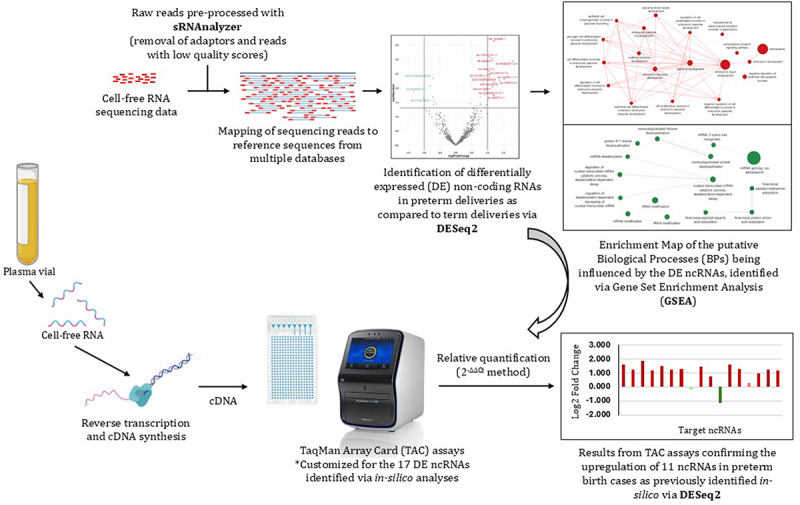

## Introduction

Preterm birth (PTB), defined as delivery before 37 weeks of gestation, leads to complications that are the leading cause of death among children under 5 years of age [1]. PTB is usually related to pathological changes including placental insufficiency, sub-clinical infections, and/or disruptions in maternal immune tolerance to pregnancy. The identification of early predictive biomarkers for PTB remains an emerging area of research, with findings that are often inconsistent. Hitherto, the focus is on finding biomarkers within 1–2% of the human genome that is translated to proteins. However, much of the transcribed DNA that produces non-coding RNAs (ncRNAs), RNAs that do not code for proteins but have critical regulatory functions [2,3], have been understudied in the context of pregnancy. ncRNAs can be categorized into two groups according to their sizes: small ncRNAs (<200 bp) and lncRNAs (>200 bp). Small ncRNAs, such as, microRNA (miRNA), piwi-interacting RNA (piRNA), small interfering RNA (siRNA), and small nucleolar RNA (snRNA) primarily act as inhibitors of gene expression, exerting negative regulatory functions [4]. miRNAs, that are 22–25 bps long, suppress the target gene expression by disrupting translation via binding the 3’ UTR region [5]. lncRNAs have been reported to have different functions that influence various biological pathways. These include transcription and translation regulation signals, interfering complementary mRNAs and miRNAs, scaffolding (binding proteins and RNAs to form functional complexes) and generating miRNAs [5,6]. Among pregnant women with preeclampsia, some long non-coding RNAs (lncRNAs) have been reported to be aberrantly expressed in the placenta [7] or maternal serum [8]. Differentially expressed (DE) lncRNAs have also been identified in human uterine tissue (myometrium, collected at caesarean section) in women with spontaneous labour at term [9]. Genome‐scale transcriptomic analysis of maternal plasma could provide an insight into how the DE ncRNAs influence the pregnancy-related biological pathways. By combining *in-silico* and *in-vitro* analyses, this study aims to elucidate the expression of ncRNAs in plasma samples from pregnant women enrolled in the AMANHI-Pakistan biorepository [10,11] cohort. The study specifically highlights significant expression differences among women between 8 and 19 weeks of gestation who have given PTB (cases) and term birth (controls). These findings provide a framework for future research into early predictive ncRNA markers for PTB and other APOs.

## Materials and methods

### Experimental groups

Jehan et al. [12] previously conducted an analytical, retrospective case-control study using cohorts from five low- and middle-income countries (LMICs) – Karachi, Pakistan; Matlab, Bangladesh; Sylhet, Bangladesh; Lusaka, Zambia; and Pemba, Tanzania – as part of the Alliance for Maternal and Newborn Health Improvement (AMANHI) [10] biorepository. In the *in-silico* phase of the current investigation, cell-free RNA sequencing (cfRNA-seq) data from 90 pregnant women (47 term and 43 preterm) [12], obtained in the aforementioned study [12], were reanalysed. Additionally, for validation through TaqMan Array Card (TAC) assays, plasma samples from 400 women enrolled in the AMANHI-Pakistan biorepository (1:1 case-control ratio) were utilized. Descriptive statistics for the clinical characteristics of the pregnancies included in the validation analysis are presented in Table 1. The number of samples from various subgroups of the preterm birth cases is presented in Table 2.

**Table 1.** Descriptive summaries of the clinical characteristics of the pregnant women considered for validation assays.

Characteristics	Term	Preterm	Total
	N = 200	N = 200	N = 400
**Maternal age, years, (mean ± SD)**	27.64 ± 4.61	26.64 ± 4.65	27.14 ± 4.65
**Maternal MUAC, cm, (mean ± SD), *n* = 349**	25.73 ± 3.73	24.78 ± 3.36	25.24 ± 3.57
**Maternal BMI, kg/m2, (mean ± SD), *n* = 395**	2.33 ± 0.73	2.12 ± 0.86	2.22 ± 0.81
**Parity**			
No Previous births	11 (5.50%)	16 (8.00%)	27 (6.75%)
1–2 Births	118 (59.00%)	122 (61.00%)	240 (60.00%)
3–5 Births	71 (35.50%)	50 (25.00%)	121 (30.25%)
>5 Births	0 (0.00%)	12 (6.00%)	12 (3.00%)
**Gravidity**			
1	0 (0.00%)	1 (0.50%)	1 (0.25%)
2	52 (26.00%)	46 (23.00%)	98 (24.50%)
3	54 (27.00%)	52 (26.00%)	106 (26.50%)
≥4	94 (47.00%)	101 (50.50%)	195 (48.75%)
**Ethnicity**			
Sindhi	52 (26.00%)	64 (32.00%)	116 (29.00%)
Punjabi	25 (12.50%)	22 (11.00%)	47 (11.75%)
Pathan	6 (3.00%)	2 (1.00%)	8 (2.00%)
Baloch	3 (1.50%)	5 (2.50%)	8 (2.00%)
Urdu Speaking	72 (36.00%)	77 (38.50%)	149 (37.25%)
Bengali	31 (15.50%)	23 (11.50%)	54 (13.50%)
Others	11 (5.50%)	7 (3.50%)	18 (4.50%)
**Hypertension during pregnancy, *n* = 393**			
Yes	25 (12.69%)	32 (16.33%)	57 (14.50%)
No	172 (87.31%)	164 (83.67%)	336 (85.50%)
**History of premature birth, *n* = 399**			
Yes	13 (6.50%)	35 (17.59%)	48 (12.03%)
No	187 (93.50%)	163 (81.91%)	350 (87.72%)
Don’t Know	0 (0.00%)	1 (0.50%)	1 (0.25%)
**Mode of delivery, *n* = 369**			
Normal Vaginal Delivery (NVD)	165 (87.30%)	125 (69.44%)	290 (78.59%)
Assisted	2 (1.06%)	1 (0.56%)	3 (0.81%)
C-section	22 (11.64%)	54 (30.00%)	76 (20.60%)
**GA at delivery, weeks, (mean ± SD)**	39.96 ± 0.65	34.80 ± 2.51	37.38 ± 3.17
**GA at sample collection, weeks, (mean ± SD)**	14.49 ± 3.48	13.14 ± 3.50	13.82 ± 3.55
**Infant sex, *n* = 353**			
Male	99 (52.94%)	89 (53.61%)	188 (53.26%)
Female	88 (47.06%)	77 (46.39%)	165 (46.74%)

**Table 2.** Number of samples from various categories of preterm birth (per the gestational age at delivery).

Preterm Categories, N = 200	n (%)
Extremely Preterm	9 (4.5%)
Very Preterm	12 (6%)
Moderate to Late Preterm	179 (89.5%)

### Ethics statement

An ethical exemption for this study was obtained from Aga Khan University’s Ethical Review Committee (AKU ERC No. 2021–3640–19709). Each participant in the initial cohort provided written informed consent, which is also valid for the current investigation.

### Participant selection criteria

The participants’ samples archived in the AMANHI-Pakistan biorepository were selected for validation within the current study based on the following criteria:

#### Inclusion

Mothers in an age range of 15–40 years residing in the study sites, i.e., Rehri Goth and Ibrahim Hyderi, Karachi, Pakistan; singleton pregnancies; samples collected at 8 to 19 weeks of gestation; spontaneous preterm births and C-sections occurring before 37 completed weeks of gestation (cases); healthy term births (controls); and mothers with recorded phenotypic data required for the current analyses.

#### Exclusion

Mothers who opted to withdraw from the current investigation or did not provide consent; pre-existing maternal diseases or comorbidities (coronary/renal/pulmonary disease, autoimmune disorders, and severe anaemia) or infections (placental/vaginal infections); mothers with medically indicated preterm deliveries; mothers with missing phenotype information required for the analyses; and multiple gestation (twins/triplets/quadruplets).

### Cell-free transcriptome sequencing data analysis

cfRNA-seq reads were retrieved from a previously published study [12]. Sequencing data, in the form of fastq files, were analysed using the RNA analysis pipeline, sRNAnalyzer [13]. Adaptor sequences were trimmed, and sequences with low-quality scores were removed. Pre-processed reads were mapped to multiple databases; human_miRNA (HMDD v3.2), human_miRNA_sub (v22.1), human_piRNA (v2.0), human_snoRNA (v1.0), human_lncRNA (v5.2), human_repSeq (RepeatMasker Edition 2018), human_subSeq (RepeatMasker Edition 2018), MirGeneDB_human_miRNA (v2.0), GtRNAdb_human_tRNA (v18.1), human_RNA (v97), human_ncRNA, nt_human_rtRNA and all_miRNA (v22.1), all_miRNA_sub (v22.1), all_rRNA_SSU (v138.1), and all_rRNA_LSU (v138.1), and miRbase (v22.1) with no mismatches allowed for the identification of potential ncRNAs. Furthermore, ncRNAs with 0 read counts in <50% of samples, or a mean read count of <20 were removed, producing a final dataset. Count Per Million (CPM) values were calculated using edgeR [14] and transformed to log2 scale for data normalization.

### Identification of PTB-Related differentially expressed ncRNAs

DESeq2 (v1.22.0) [15] was used to identify significant differences in expression of ncRNAs between case/control groups if they exhibited a Benjamini‐Hochberg (BH) adjusted *p* value of <0.05 and a log2FC of >1 or <−1. The analysis was adjusted for multiple testing using the BH procedure to control the false discovery rate (FDR). Volcano plot was generated by ggplot2 (v3.4.4) package in R to represent the combined impact of statistical significance (BH-adjusted q value <0.05) and expression magnitude (log2FC >1 or < −1), aiding in the identification of prominent alterations in the expression of upregulated and downregulated ncRNAs.

### Gene set enrichment analysis (GSEA)

Identified from DeSeq2, significantly DE ncRNAs (with BH FDR adjusted *p* value set to <0.05) were considered for GSEA using *g:Profiler* [16]. Gene Ontology (GO) terms were classified into biological processes (BPs), molecular functions (MFs) and KEGG terms along with their *p* values. Involvement of significant GO terms in various pathways was visualized using Cytoscape 3.10.1 [17]. The interactions were identified using EnrichmentMap (3.3.6) app of Cytoscape.

### Annotation of ncRNAs

We adopted a multi-dimensional approach to annotate ncRNAs. The NONCODE database, designed for the annotation and analysis of lncRNAs, provided a foundation for our study. LNCipedia is incorporated to offer a specialized focus on human lncRNA transcripts and genes. The GeneCards database, a searchable resource that integrates data from ~150 web sources, provided a comprehensive overview of annotated and predicted genes in both groups.

In addition to these databases, various metrics are employed to enhance the depth of ncRNA annotation. RNAcentral facilitated access to a comprehensive set of non-coding RNA sequences, representing diverse species. Exon numbers and sequence ontology (SO) terms contributed to understanding the structural components and genomic context of ncRNAs. The Coding-Non-Coding Index (CNCI) score, GeneCards Inferred Functionality Scores (GIFtS) algorithm, and PhyloCSF score provided insights into coding potential, functionality, and evolutionary conservation.

Furthermore, we also assessed coding potential of ncRNAs using Coding-Potential Assessment Tool (CPAT) Coding Probability, Pride Reprocessing, and Lee Translational Initiation sites. These classifiers utilize sequence features to distinguish between protein-coding and non-coding RNAs, contributing to an insightful understanding of ncRNA function. Locus Conservation evaluates the degree of similarity across different genetic loci, while the In Stringent Set concept sheds light on regulatory mechanisms during bacterial stringent responses (Supplementary Dataset S1, https://doi.org/10.6084/m9.figshare.27079126).

### Experimental validation of differentially expressed ncRNAs by rt-qPCR array

For the *in-vitro* TAC assays and targeted ncRNAs’ expression analyses, RNAs were extracted from 400 maternal plasma samples (sourced from the AMANHI-Pakistan cohort [10]) using an automated magnetic bead – based extractor, MagNA Pure 24 systems, and a compatible nucleic acid isolation kit I (Roche Life Sciences, Mannheim, Germany) according to manufacturer’s recommendations. The extracted RNA samples were checked for quality via Nanodrop spectrophotometer by measuring the absorbance at specific wavelengths; 260 nm, 280 nm and 230 nm. TAC assay was customized by the manufacturer as per the shared sequences of the target ncRNAs (lnc-RABGEF1–2:4, lnc-SBDS-3:1, lnc-VAMP1–1:13, lnc-COMMD10–10:1, lnc-ABAT-2:1, lnc-DHX15–1:2, lnc-VN1R2–1:1, lnc-OR4F4–4:3, lnc-TNFSF10–1:1, lnc-MEST-4:1, NR_002989.1, ENST00000384674, hsa-mir-3648–1, piR-hsa -28,755, lnc-FCGR1B–6:1, ENST00000579700, lnc-ADAM32–2:1), and performed as described previously [18]. Briefly, PCR reaction mix was prepared using RNA and AgPath One Step RT-PCR kit (Thermo Fisher), which was loaded on the card, followed by spinning, sealing, and running on Applied Biosystem QuantStudio 7 system. All samples were analysed in duplicates and mean CT values were calculated. The fold change in expression of the target ncRNA relative to the housekeeping gene, human *GAPDH* (GAPDH-Hs99999905_m1), was calculated using the 2^−ΔΔCt^ method.

### Statistical analyses

The fold changes in ncRNA expression (calculated from TAC assay results) in women delivering preterm and term were compared using 2-tailed two-sample *T test*. Results are expressed as log2 Fold Change (log2FC) in expression of ncRNAs relative to the housekeeping gene (GAPDH-Hs99999905_m1). These statistical tests were carried out using MS Excel, and the calculations are presented in the Supplementary Dataset S2; https://doi.org/10.6084/m9.figshare.27079126).

## Results

**Figure 1.**
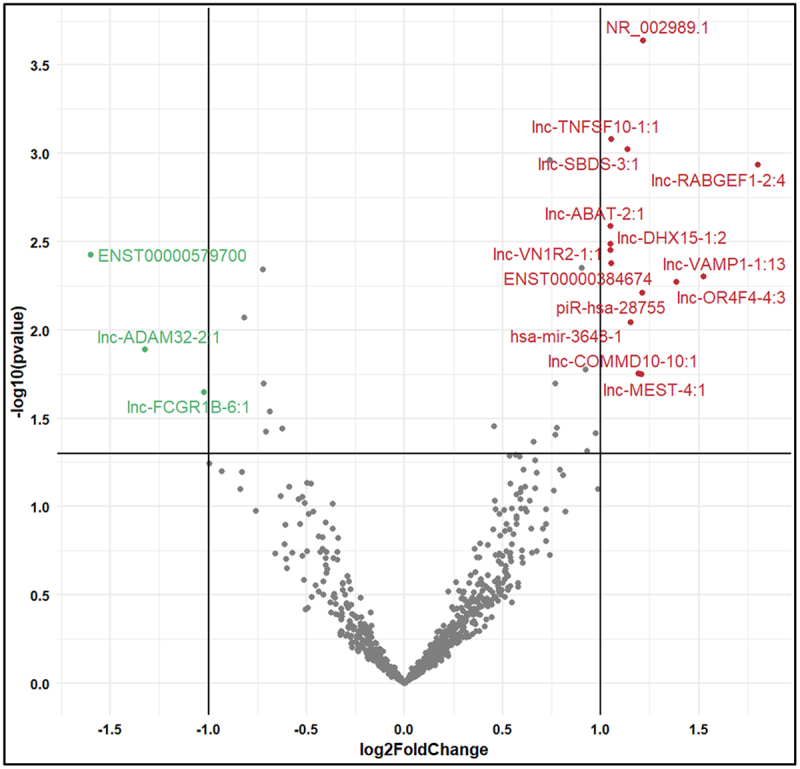
Volcano plot of DE ncRNAs in PTB compared to term birth. The x-axis represents the log2 fold change (log2FC) in ncRNA expression between PTB and term birth. A positive value indicates upregulation in PTB cases, and a negative value indicates downregulation. The y-axis represents the -log10(p-value), where the p-value is a statistical measure of the significance of the observed difference in expression between the two groups. Higher -log10(p-value) indicates stronger statistical significance. The red dots represent upregulated ncRNAs in PTB cases compared to term birth controls. The green dots represent downregulated ncRNAs in PTB cases compared to term delivery controls.

The *in-silico* analyses revealed 10 lncRNAs, 1 miRNA, 2 snoRNAs and 1 piRNA as upregulated, while 3 lncRNAs as downregulated in PTB cases as compared with the expression in term birth controls [per the recorded log2 fold change (Log2FC) from DESeq2 pipeline] from a total of 2,122 ncRNAs. These DE ncRNAs, listed in Table 3, and illustrated on a volcano plot (Figure 1), served as an important foundation for subsequent analysis.

**Table 3.** Upregulated and downregulated ncRNAs in PTB, identified by *in-silico* analysis (DeSeq2).

S. No.	Upregulated ncRNAs		
	Transcript ID	log2FC	*P* value
1.	lnc-RABGEF1–2:4	1.803	1.1 × 10^−3^
2.	lnc-SBDS-3:1	1.141	9.4 × 10^−4^
3.	lnc-VAMP1–1:13	1.524	5.0 × 10^−3^
4.	lnc-COMMD10–10:1	1.194	1.7 × 10^−2^
5.	lnc-ABAT-2:1	1.031	2.5 × 10^−2^
6.	lnc-DHX15–1:2	1.052	3.2 × 10^−2^
7.	lnc-VN1R2–1:1	1.010	3.5 × 10^−2^
8.	lnc-OR4F4–4:3	1.386	5.3 × 10^−2^
9.	lnc-TNFSF10–1:1	1.004	8.3 × 10^−4^
10.	lnc-MEST-4:1	1.209	1.7 × 10^−2^
11.	NR_002989.1	1.217	2.2 × 10^−4^
12.	ENST00000384674	1.036	4.2 × 10^−3^
13.	hsa-mir-3648–1	1.152	9.0 × 10^−3^
14.	piR-hsa -28,755	1.214	6.1 × 10^−3^
S. No.	Downregulated ncRNAs		
	Transcript ID	log2FC	*P* value
1.	lnc-FCGR1B–6:1	−1.023	2.2 × 10^−2^
2.	ENST00000579700	−1.600	3.7 × 10^−3^
3.	lnc-ADAM32–2:1	−1.324	1.2 × 10^−2^

**Figure 2.**
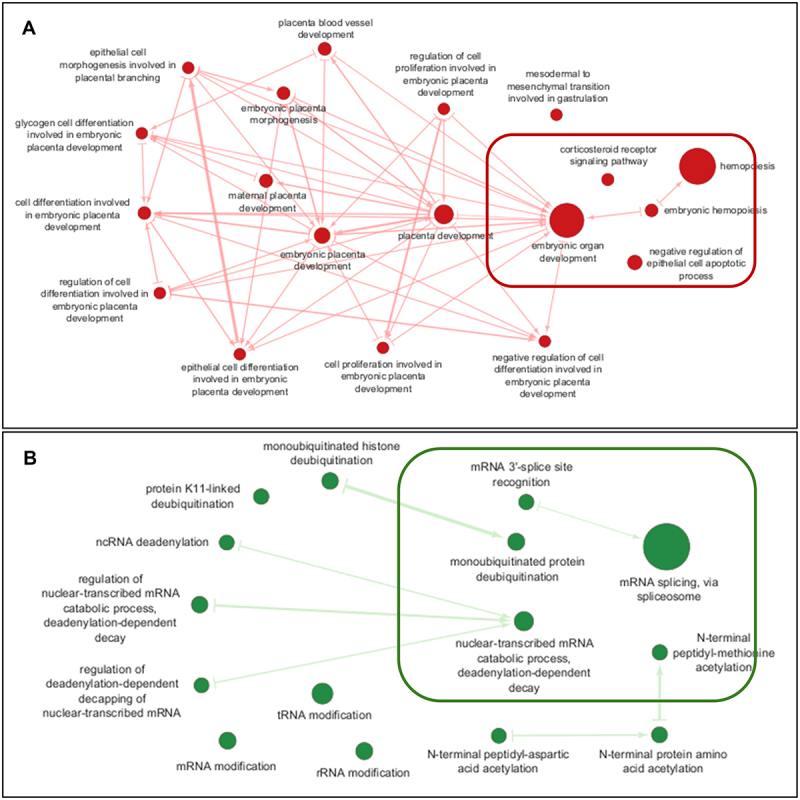
Enrichment map of top GO BPs from the upregulated and downregulated ncRNA gene lists. The nodes represent gene sets, and the edges represent the overlap between gene sets. The size of a node corresponds to the number of genes in the gene set. Edge size corresponds to the number of genes that overlap between two connected gene sets. The red circles in **A** highlight the GO biological processes (BPs) from the upregulated ncRNA gene list. The green circles in **B** highlight the GO BPs from the downregulated ncRNA gene list. Highlighted in the rounded rectangles are the top 5 GO BPs from each gene list.

Gene lists containing 14 upregulated ncRNAs (along with 30 transcripts) and 3 downregulated ncRNAs (along with 19 transcripts) were submitted to *g:Profiler* for the identification of Gene Ontology (GO) terms. Eight hundred and eighty Biological Processes (BPs) were determined from the upregulated gene list, and 607 BPs were identified as downregulated. Top 5 GO terms for BPs from up/downregulated gene lists were visualized using Cytoscape. These top 5 BPs (Table 4) influenced by the DE ncRNAs are highlighted in the enrichment map (Figure 2).

**Table 4.** Top 5 biological processes (BPs) for DE ncRnas.

Upregulated Gene List		Downregulated Gene List	
BP Term	*P* value	BP Term	*P* value
Embryonic Hemopoiesis (GO:0035162)	2.5 × 10^−4^	Nuclear-transcribed mRNA Catabolic Process, Deadenylation-dependent Decay (GO:0000288)	2.1 × 10^−3^
Negative Regulation of Epithelial Cell Apoptotic Process (GO:1904036)	1.8 × 10^−3^	mRNA Splicing, via Spliceosome (GO:0000398)	2.8 × 10^−3^
Embryonic Organ Development (GO:0048568)	6.0 × 10^−3^	N-terminal Peptidyl-Methionine Acetylation (GO:0017196)	6.5 × 10^−3^
Hemopoiesis (GO:0030097)	1.2 × 10^−2^	Monoubiquitinated Protein Deubiquitination (GO:0035520)	6.58 × 10^−3^
Corticosteroid Receptor Signaling Pathway (GO:0031958)	1.3 × 10^−2^	mRNA 3’-splice Site Recognition (GO:0000389)	6.5 × 10^−3^

**Figure 3.**
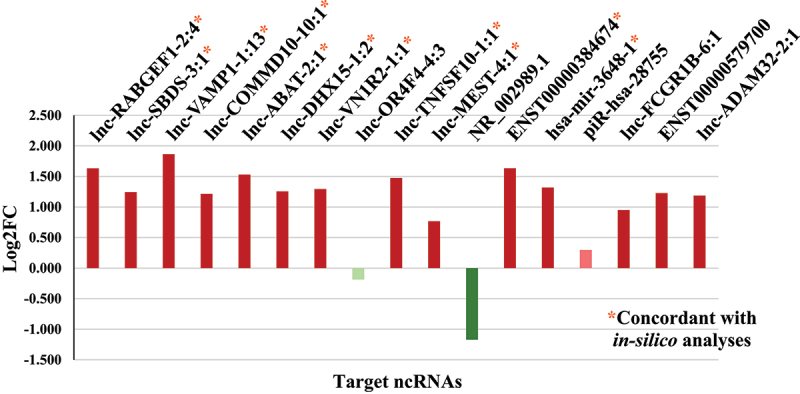
Bar chart showing log2 fold change (log2FC) in the expression of each targeted ncRNA relative to the reference gene (*GAPDH*) in PTB compared with term birth. The x-axis represents the targeted ncRnas. The y-axis represents the log2 fold change (log2FC) in the expression of targeted ncRNAs relative to the reference gene (GAPDH). A positive value indicates upregulation in preterm cases compared to term delivery controls, and a negative value indicates downregulation. The fold changes in targeted ncRNA expression were calculated using the ct values recorded from the TAC assays/rt-qPCR and the 2^− ΔΔCt^ method. The red and green bars reflect the upregulated and downregulated ncRnas, respectively. Dark-coloured bars show the ncRNAs that significantly differed in expression, while the light-coloured bars show the ncRNAs that did not significantly differ in expression, in PTB cases compared with term controls.

Results from the TaqMan Array Card (TAC) assay (Figure 3) showed statistically significant upregulation of 14 ncRNAs in women with PTB compared to the women with term birth. The calculated Log2FC in expressions of 11 ncRNAs; lnc-RABGEF1–2:4 (*p* value: 0.002), lnc-SBDS-3:1 (*p* value: 0.001), lnc-VAMP1–1:13 (*p* value: 0.003), lnc-COMMD10–10:1 (*p* value: 0.0005), lnc-ABAT-2:1 (*p* value: 0.003), lnc-DHX15–1:2 *p* value: 0.001), lnc-VN1R2–1:1 (*p* value: 0.0004), lnc-TNFSF10–1:1 (*p* value: 0.007), lnc-MEST-4:1 (*p* value: 0.025), ENST00000384674 (*p* value: 0.004), and hsa-mir-3648–1 (*p* value: 0.035) were observed to be concordant with the results obtained from the *in-silico* analyses (Supplementary Dataset S2; https://doi.org/10.6084/m9.figshare.27079126).

## Discussion

Over 75% of the human genome undergoes transcription into ncRNAs. With the recent reports of high expression of ncRNAs in the placenta during parturition and the identification of placenta-derived ncRNAs in maternal plasma [19], it could be hypothesized that these ncRNAs play pivotal roles in placentation and modulating various biological pathways associated with pregnancy and embryonic development. The current nested case-control study was designed to identify differential expressions of cell-free plasma ncRNAs in pregnant women that may be associated with the risk of PTB.

Of the 17 DE ncRNAs we identified via the *in-silico* analyses, the majority have not been annotated to date. However, the regulatory roles of some of these ncRNAs have been reported in the literature after experimental validation/s. hsa-mir-3648–1 has been shown to target and downregulate Adenomatous polyposis coli 2 (*APC2*) leading to increased cell proliferation [20]. Therefore, it is a rational biological plausibility that cell proliferation is likely to be altered in PTB cases via *APC2* downregulation.

Embryonic Hemopoiesis (GO: 0035162), the most statistically significant BP for the upregulated gene list, is linked to embryonic organ development (Figure 2A) which agrees with the findings from previous research evidencing the role of ncRNAs in haemopoietic differentiation [21]. A study has shown that the expression of lncRNA (EGO – Eosinophil Granule Ontogeny/EGOT), in the bone marrow CD34+ cells and bone marrow mononuclear cells (BMMCs), is involved in regulating differentiation in the eosinophil lineage and is specific to Bone marrow CD34+ cells and bone marrow mononuclear cells (BMMCs) [22]. Moreover, several studies have reported that the lncRNA (H19), expressed from the maternal allele in extra-embryonic cell types, is associated with haematopoietic stem cell quiescence and controls *IGF*2 expression and foetus growth [23,24].

In the top 5 BPs for the downregulated gene list, Nuclear-transcribed mRNA Catabolic Process, Deadenylation-dependent Decay (GO:0000288) was identified as the most significant BP. The enrichment map (Figure 2B) shows that Nuclear-transcribed mRNA Catabolic Process, Deadenylation-dependent Decay is connected to 3 other BPs; ncRNA deadenylation, regulation of nuclear-transcribed mRNA catabolic process, deadenylation-dependent decay, and ‘regulation of deadenylation-dependent decapping of nuclear-transcribed mRNA. This is suggestive of the role of these DE ncRNA in biological pathways that may lead to PTB.

Moreover, the 17 PTB-associated DE ncRNAs identified via *in-silico* analyses (Table 3) were validated in the subsequent TAC assays. Comparative analyses revealed 11 ncRNAs (Figure 3 and Supplementary Dataset S2; https://doi.org/10.6084/m9.figshare.27079126) that were DE in PTB cases, exhibiting a log2 FC of >1 or <−1. A recent study on the Chinese population has reported high expression of lncRNA SNHG29, which may be associated with cell senescence, in the placenta of women who delivered preterm with labour when compared with women who delivered preterm without labour and full-term birth with/without labour, quantified using Real-Time quantitative PCR (RT-qPCR) [25]. However, SNHG29 was not identified as DE lncRNA in the plasma samples from the pregnant women enrolled in this study. The absence of the stimulus causing the high expression of lncRNA SNHG29 in the studied population may account for this discrepancy.

The findings from this study align with existing literature, signifying that profiling ncRNAs in plasma during the early stages of pregnancy, such as the first trimester and early second trimester, has the potential to identify risks associated with spontaneous PTB. Further investigations with larger sample sizes are needed to validate the associations of DE ncRNAs with PTB and other APOs like preeclampsia (PE) and Intrauterine Growth Restriction (IUGR). Multiple studies [26,27] have advocated the role of ncRNAs in reproductive health of women and pregnancy outcomes, but the limited understanding of functional annotations impedes diagnosis, and treatment. Advanced technologies and collaborative efforts are essential for uncovering the potential of ncRNAs and improving maternal and fetal health.

## Limitations

The samples analysed in this study were retrieved from a previously established AMANHI-Pakistan biorepository and the sample size was determined using convenience sampling, a nonprobability sampling method based on the ease of access and availability of the sample at a specific time. This sample may not be representative of the broader population. The focus on the samples from Pakistani population may limit the generalizability of the findings to other regions or settings. Hence, further studies on larger independent cohorts from other LMICs could validate the results obtained via this study. Moreover, as the participants in the AMANHI biorepository got enrolled during 8–19 weeks of gestation, a narrower window of gestational age at sample collection in future studies could further refine the findings. Secondly, while GSEA and GO terms provided some insights into the biological mechanisms being affected by the DE ncRNAs, the functional interpretations are dependent on current databases, which lack annotations of majority of the ncRNAs identified to date. Functional annotations of the various types of ncRNAs are much needed to fully understand the cascades of biological processes that are altered in different APOs. Lastly, this study analysed the samples collected at one time point only, not including longitudinal tracking of ncRNA expression across gestational phases, which could provide additional insight into the dynamic changes over time.

## Conclusion

The current investigation identified a distinct profile of differentially expressed ncRNAs, including lncRNAs, miRNAs, snoRNAs, and piRNAs, associated with PTB compared to term births. The upregulation of specific lncRNAs, such as lnc-RABGEF1–2:4, lnc-SBDS-3:1, and lnc-VAMP1–1:13, along with the miRNA has-mirx-3648–1, suggests their potential involvement in PTB pathogenesis. Further functional characterization of these ncRNAs could elucidate their precise roles in preterm labour and identify potential therapeutic targets.

## Supplementary Material

Supplemental Material

## Data Availability

Data will be available upon request.
