# Supplementary figures and images for: Identification of differentially expressed non-coding RNAs in the plasma of women with preterm birth
Source: RNA Biol. 2025 Jan 13;22(1):1–8. doi: 10.1080/15476286.2024.2449278 (PMC11730358; doi:10.1080/15476286.2024.2449278)

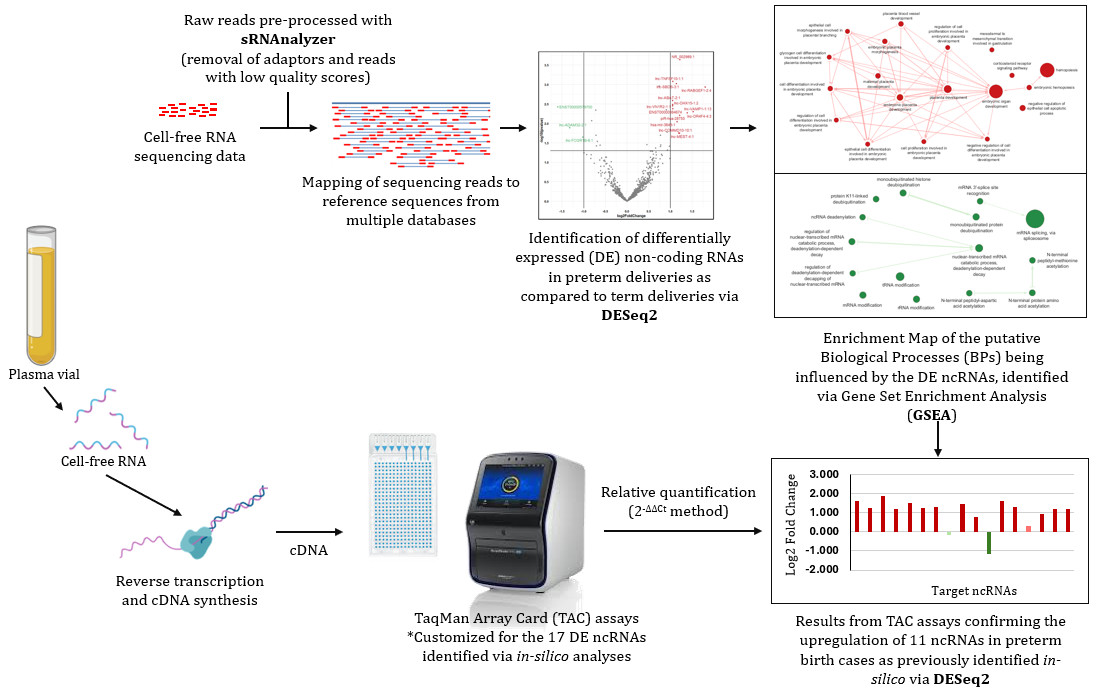

Supplement: Supplemental Material [file KRNB_A_2449278_SM7110.zip › grphcl_abstrct_AKU_ncRNA.jpg]
